# Impact of a training program on the surveillance of *Clostridioides difficile* infection

**DOI:** 10.1017/S0950268819001080

**Published:** 2019-07-02

**Authors:** N. Sopena, N. Freixas, F. Bella, J. Pérez, A. Hornero, E. Limon, F. Gudiol, M. Pujol, the VINCat Clostridioides difficile study group

**Affiliations:** 1 Infectious Diseases Department, Hospital Universitari Germans Trias i Pujol, Badalona, Spain; 2 VINCat Program, Catalonia, Spain; 3 Infection Control Team, Hospital Universitari Mútua Terrassa, Terrassa, Spain; 4 Infectious Diseases Unit, Hospital de Terrassa, Terrassa, Spain; 5 Microbiology Laboratory, CATLAB, Viladecavalls, Spain; 6 Infection Control Team, Hospital Universitari de Bellvitge-IDIBELL, L'Hospitalet de Llobregat, Barcelona, Spain; 7 Nursing School of the Medicine and Health Sciences Faculty, Universitat de Barcelona; 8 Infectious Diseases Department, Hospital Universitari de Bellvitge-IDIBELL, L'Hospitalet de Llobregat, Barcelona, Spain

**Keywords:** *Clostridioides difficile*, *Clostridium difficile*, infection prevention, medical education, surveillance

## Abstract

A high degree of vigilance and appropriate diagnostic methods are required to detect *Clostridioides difficile* infection (CDI). We studied the effectiveness of a multimodal training program for improving CDI surveillance and prevention. Between 2011 and 2016, this program was made available to healthcare staff of acute care hospitals in Catalonia. The program included an online course, two face-to-face workshops and dissemination of recommendations on prevention and diagnosis. Adherence to the recommendations was evaluated through surveys administered to the infection control teams at the 38 participating hospitals. The incidence of CDI increased from 2.20 cases/10 000 patient-days in 2011 to 3.41 in 2016 (*P* < 0.001). The number of hospitals that applied an optimal diagnostic algorithm rose from 32.0% to 71.1% (*P* = 0.002). Hospitals that applied an optimal diagnostic algorithm reported a higher overall incidence of CDI (3.62 *vs.* 1.92, *P* < 0.001), and hospitals that were more active in searching for cases reported higher rates of hospital-acquired CDI (1.76 *vs.* 0.84, *P* < 0.001). The results suggest that the application of a multimodal training strategy was associated with a significant rise in the reporting of CDI, as well as with an increase in the application of the optimal diagnostic algorithm.

## Introduction

*Clostridioides difficile* (formerly *Clostridium difficile*) is the most frequent cause of healthcare-related infectious diarrhoea, which mainly affects elderly patients presenting risk factors such as the use of antibiotics [1]. Patients are exposed to spores of the organism through contact with the hands of the health personnel or the hospital environment [2]. The diagnosis of *Clostridioides difficile* infection (CDI) is based on clinical suspicion and on the application of adequate laboratory techniques. In recent years, microbiological diagnosis has been enhanced by the incorporation of more sensitive tests in diagnostic algorithms such as molecular techniques [3]. The prevention of CDI in the healthcare setting depends on early diagnosis and the application of a set of measures including hand hygiene and the cleaning and disinfection of surfaces, and the proper use of antibiotics [1].

In developed countries, the incidence of CDI, including CDI of nosocomial origin, has risen in recent years [4–6]. However, according to a study conducted in Europe between 2011 and 2013, its incidence varies across countries from 0 to 36.3/10 000 patient-days [7], due in part to the low diagnostic suspicion and the application of suboptimal diagnostic methods; in fact, in that study, only 52% of hospitals used an optimal diagnostic algorithm [7]. The training of health professionals is fundamental in CDI prevention programs in order to improve the detection of cases and to increase compliance with preventive measures [8].

The aim of this study was to evaluate the effectiveness of a multimodal training program in improving the surveillance and prevention of CDI in hospitals participating in the nosocomial infection surveillance program in Catalonia (VINCat) [9].

## Methods

This multicentre, prospective, quasi-experimental study designed by the VINCat program's CDI study group assessed the multimodal training program by comparing the baseline phase (2011) with the post-training phase (2016). The items evaluated were the evolution of the number of hospitals participating in the CDI surveillance program, the evolution of the CDI rates reported, the percentage of centres using appropriate CDI diagnostic methodology and the adequacy of the prevention measures applied at each centre.

### Study setting

VINCat is a nosocomial infection surveillance and control program which has been in operation at acute care hospitals in Catalonia since 2006 [9]. The present study was carried out in the hospitals in the VINCat program that reports cases of CDI. All these hospitals have multidisciplinary infection control teams, and they are stratified into three groups according to size: group 1 (more than 500 beds), group 2 (between 200 and 500 beds) and group 3 (fewer than 200 beds).

### Intervention

Between 2011 and 2015, a multimodal training program was implemented, which comprised the following elements:

1. An online course for physicians, microbiologists and nursing staff at Catalan hospitals. The course consisted of five parts: epidemiology and clinical manifestations of CDI; microbiological and non-microbiological diagnosis; transmission mechanisms, surveillance and prevention measures; treatment of diarrhoea associated with *C. difficile*; and CDI in special populations. Each section included a text, summaries, algorithms, images and recommendations for further reading, and ended with a test that participants had to complete before proceeding to the next section.
2. Participation was voluntary. Healthcare professionals from all hospitals in Catalonia could participate in the course, regardless of whether they reported or not cases of CDI to the VINCat program. The course was promoted by means of leaflets, e-mails to the coordinators of the centres and members of the Catalan Association of Infection Control Nurses, at congresses and scientific workshops and on the VINCat program website.
3. Participants could access a student and tutor forum where they could ask and answer questions. Immediately after the study period, they were asked to answer a multi-choice quiz with 10–12 questions, each one with a correct answer and three distractors: one point was awarded for a correct answer and zero points for an incorrect or no answer. The result was provided at the end of the test. The course was offered five times; in all, 890 health professionals participated.
4. Face-to-face training. Two workshops were conducted for the staff of the hospitals (physicians, microbiologists and nurses) participating in the VINCat program: the first in 2011, attended by 129 participants, and the second in 2013, attended by 149 participants. The workshops included feedback on the incidence rates of CDI at the participating hospitals, measures to prevent transmission and discussion of practical cases with regard to surveillance criteria and microbiological diagnosis.
5. Preparation (and dissemination via the VINCat program website) of recommended transmission prevention measures, the microbiological diagnostic algorithm, information on CDI for patients and relatives, a checklist for cleaning and disinfection of surfaces possibly contaminated by *C. difficile*, and recommended further reading.
6. Participants' satisfaction survey regarding the training activities, evaluating: online training, the diagnostic algorithm, recommended precautions, information for the patient, the checklist of cleaning and disinfection of surfaces, recommended bibliography and feedback on the incidence rates reported by hospitals. A numerical scale from 1 to 5 was used (1: not very useful, 5: very useful), with scores 4 and 5 indicating a high level of satisfaction.

### CDI surveillance

Cases of CDI were followed up continuously from 2011 to 2016, using the methodology previously defined by the VINCat program.

Population under surveillance: adults over 18 years of age attended in any hospital sector (wards, emergency services, outpatient consultations, etc.) who met the definition of CDI. Asymptomatic patients were excluded, even if they were carriers of a toxin-producing strain, as were patients with a history of CDI and those at specific palliative care or convalescence units.

Definition of CDI: a patient with acute diarrhoea (defined as three or more unformed bowel movements in 24 h), or toxic megacolon without another known cause, plus one of the following: (1) stool sample with a toxin A- or B-positive laboratory result for *C. difficile*, or detection of genes that encodes toxin by molecular testing; (2) endoscopic, surgical or histological examination confirming the diagnosis of pseudomembranous colitis.

### Classification according to the site of acquisition of diarrhoea

Hospital-acquired CDI: infection identified >48 h after admission to the hospital and before discharge.

Non-nosocomial healthcare-related CDI: infection starting in the community or within 48 h of admission, in patients admitted to a health centre (hospital, nursing home or community health centre) in the 4 weeks prior to the onset of symptoms.

Community-acquired CDI: infection starting in the community or within 48 h of admission, with no admission to a health centre in the last 4 weeks.

Incidence rates were expressed in the number of cases per 10 000 patient-days.

The coordinating centre provided feedback to each hospital on an annual basis, reporting their rates and comparing them with the rest of the hospitals (means, medians and percentiles) and according to the size of the hospital.

### Survey of adherence to recommendations

To assess adherence to the recommended diagnostic methods and preventive measures, a self-administered questionnaire was used in 2011 (baseline measurement) and 2016 (post-training measurement). A group of infection control experts validated the content and structure of the questionnaire for comprehensibility and consistency. The questionnaire had two sections, the first with nine questions on diagnostic aspects and the second with seven questions on preventive measures. The 2016 questionnaire also incorporated the satisfaction survey on the different training activities and instruments provided. The questionnaire was distributed electronically by the coordinating centre, and the results were entered into a database for further analysis. The diagnostic methodology was considered optimal when an algorithm including two or more detection methods for toxigenic *C. difficile* was applied. The most frequently used algorithm was the one that detected the GDH enzyme by enzyme-linked immunosorbent assay as a screening method, followed by the detection of the A/B toxin either by enzyme-linked immunosorbent assay or by molecular methods. Search intensity (i.e. the attention paid to identifying CDI cases) was considered adequate when the presence of toxigenic *C. difficile* was investigated in more than 80% of cases of nosocomial diarrhoea. The survey was answered by the person in charge of the infection control team at each hospital, after discussing it with the team members (clinicians, nurses and microbiologists).

### Statistical analysis

CDI incidence rates are expressed as the number of CDI cases per 10 000 patient-days, with 95% confidence intervals (95% CI). Descriptive analysis was performed using counts and percentages for categorical and stratified continuous variables.

To evaluate the impact of the training program, we compared the number of participating hospitals and the incidence density rate of CDI reported by the hospitals over the period between 2011 and 2016. To assess the impact of the intervention on diagnostic methods and prevention measures, we compared the compliance rates observed in the initial and the post-training surveys. In 2016, at the end of the study, CDI incidence rates were compared in hospitals that applied an optimal diagnostic methodology and in hospitals that applied a suboptimal methodology. Likewise, the rates of hospital-acquired CDI were compared between hospitals with an adequate search intensity and hospitals with a lower intensity. For the comparison of proportions, the *χ*^2^ test or Fisher's exact test was used, as appropriate. For the comparison of the annual CDI incidence rates, the Cochran–Armitage trend test was used. A *P* value of 0.05 or less was considered statistically significant. For the statistical analysis, the EPIDAT V 3.1 program was used.

## Results

The number of hospitals reporting CDI rates to the VINCat program rose from 29 in 2011 to 47 in 2016. Of these, 25 centres completed the survey in 2011 (seven with >500 beds, 13 with 200–500 beds and five with <200 beds) and 38 centres in 2016 (five with >500 beds, 13 with 200–500 beds and 20 with <200 beds).

During the 6 years of the study, 4931 cases of CDI were reported, of which 2196 (44.5%) were hospital-acquired, 1518 (30.8%) non-nosocomial healthcare-related and 1217 (24.7%) community-acquired. Table 1 shows the evolution of the reported incidence of CDI. There was a significant and constant increase in the overall incidence reported by the group of hospitals between 2011 and 2016. This increase was also observed for hospital-acquired CDI, non-nosocomial healthcare-related CDI and community-acquired CDI. This constant and significant upward trend was also observed when only the hospitals that reported cases since 2011 were considered (Table 2).

**Table 1.** Evolution of rates of *Clostridioides difficile* infection and of the number of hospitals participating in the surveillance program

	2011 (29 hospitals) MI^a^ (95%CI)	2012 (34 hospitals) MI^a^ (95%CI)	2013 (37 hospitals) MI^a^ (95%CI)	2014 (43 hospitals) MI^a^ (95%CI)	2015 (45 hospitals) MI^a^ (95%CI)	2016 (47 hospitals) MI^a^ (95%CI)	*P* value
Overall CDI	2.20 (2.00–2.40)	2.38 (2.19–2.57)	2.65 (2.45–2.85)	3.34 (3.13–3.54)	3.62 (3.41–3.83)	3.41 (3.21–3.61)	<0.001
Hospital-acquired CDI	0.98 (0.85–1.11)	1.11 (0.97–1.24)	1.16 (1.02–1.29)	1.49 (1.35–1.62)	1.45 (1.41–1.68)	1.57 (1.43–1.70)	0.001
Non-nosocomial healthcare-related CDI	0.77 (0.65–0.89)	0.72 (0.61–0.82)	0.87 (0.75–0.98)	1.07 (0.95–1.18)	1.09 (0.97–1.20)	0.95 (0.84–1.06)	0.028
Community-acquired CDI	0.45 (0.36–0.54)	0.55 (0.46–0.65)	0.63 (0.53–0.72)	0.78 (0.68–0.89)	0.99 (0.88–1.10)	0.89 (0.79–0.99)	0.001

CDI, *Clostridioides difficile* infection.

^a^Mean incidence: cases/10 000 patient-days.

**Table 2.** Evolution of rates of *Clostridioides difficile* infection in the 29 hospitals participating in the surveillance program from 2011 to 2016

	2011 MI^a^ (95%CI)	2012 MI^a^ (95%CI)	2013 MI^a^ (95%CI)	2014 MI^a^ (95%CI)	2015 MI^a^ (95%CI)	2016 MI^a^ (95%CI)	*P* value
Overall CDI	2.26 (2.06–2.47)	2.49 (2.28–2.71)	2.72 (2.49–2.96)	3.30 (3.06–3.55)	3.54 (3.28–3.79)	3.27 (3.03–3.51)	<0.001
Hospital-acquired CDI	1.01 (0.88–1.15)	1.14 (1.00–1.29)	1.24 (1.09–1.40)	1.54 (1.37–1.71)	1.58 (1.41–1.75)	1.47 (1.30–1.63)	0.003
Non-nosocomial healthcare-related CDI	0.78 (0.66–0.90)	0.79 (0.67–0.91)	0.91 (0.77–1.04)	1.02 (0.88–1.15)	1.09 (0.95–1.23)	0.98 (0.85–1.11)	0.028
Community-acquired CDI	0.47 (0.37–0.56)	0.56 (0.46–0.67)	0.57 (0.47–0.68)	0.76 (0.64–0.87)	0.86 (0.74–0.99)	0.82 (0.70–0.94)	0.004

CDI, *Clostridioides difficile* infection.

^a^Mean incidence: cases/10 000 patient-days.

Table 3 shows the results of the surveys on CDI diagnosis and prevention. With regard to the diagnostic methods, a significant increase in the application of the optimal diagnostic algorithm was observed. There was an increase in the search intensity for CDI in patients admitted from nursing homes or from community health centres and in patients with community-acquired diarrhoeas, as well as greater adherence to most of the preventive measures recommended, although the differences did not reach statistical significance.

**Table 3.** Survey of diagnosis and preventive measures (2011 *vs*. 2016)

	2011 (25 hospitals)	2016 (38 hospitals)	
*N* (%)	*N* (%)	*P* value
CDI study in >80% of episodes of hospital-onset diarrhoea	16 (64.0)	23 (60.5)	0.78
CDI study in >50% of episodes of community-onset diarrhoea with history of antibiotic treatment	18 (72.0)	31 (81.2)	0.37
CDI study in >50% of episodes of diarrhoea in patients admitted from nursing homes or community healthcare centres	9 (36.0)	22 (57.9)	0.08
Only liquid or semiliquid faeces processed for CDI study	25 (100)	37 (97.4)	1.00
All the samples for CDI study sent to the laboratory in a bottle (not in a swab)	24 (96.0)	37 (97.4)	1.00
Optimal microbiological diagnostic methodology^a^	8 (32.0)	27 (71.1)	0.002
Availability of centre's own protocol on diagnosis and preventive measures	15 (60.0)	25 (65.8)	0.64
Daily notification by the Microbiology Service of positive results for *C. difficile*	23 (92.0)	35 (92.1)	1.00
Introduction of contact precautions in all cases of CDI	25 (100)	38 (100)	NS
Recommendation to wash hands with soap and water instead of alcohol	23 (92.0)	38 (100)	0.15
Duration of precautions at least 48–72 h after the end of diarrhoea	21 (84.0)	34 (89.5)	0.70
Consideration of whether to suspend antibiotic treatment	25 (100)	38 (100)	NS
Consideration of whether to suspend antacid treatment	1 (4.0)	9 (23.7)	0.07
No post-treatment microbiological control performed in patients diagnosed with CDI	19 (76.0)	34 (89.5)	0.17

CDI, *Clostridioides difficile* infection.

NS, not significant.

^a^The diagnostic method was considered optimal when a diagnostic algorithm including two or more methods of detection of toxigenic *C. difficile* was applied.

Focusing solely on the seven hospitals that already used an optimal diagnostic algorithm in 2011 and presented an adequate search intensity, the overall incidence of CDI did not change significantly over the study period (from 3.51 cases/10 000 patient-days (95% CI 3.09–3.92) in 2011 to 2.96 (95% CI 2.60–3.32) in 2016, *P* = 0.054); nor were there significant changes in the incidence of hospital-acquired CDI (from 1.74 (95% CI 1.45–2.03) in 2011 to 1.43 (95% CI 1.18–1-68) in 2016, *P* = 0.122).

In 2016, the incidence of CDI reported by hospitals that used an optimal diagnostic methodology (3.62 cases/10 000 patient-days, 95% CI 3.34–3.90) was higher than that reported by hospitals that used a suboptimal methodology (1.92 cases/10 000 patient-days, 95% CI 1.42–2.41, *P* < 0.001). Likewise, the incidence of hospital-acquired CDI reported by hospitals with an adequate search intensity (1.76 cases/10 000 patient-days, 95% CI 1.55–1.97) was higher than that reported by the hospitals with a lower search intensity (0.84 cases/10 000 patient-days, 95% CI 0.61–1.08, *P* < 0.001).

Thirty-eight hospitals answered the survey on the degree of satisfaction among health staff in relation to the training tools and documents provided. The rates of responses with a high degree of satisfaction (4 or 5 on a scale of 1 to 5) were 97.2% for the online training course, 94.1% for the annual feedback on the centre's results, 94.1% for the recommendations for preventive measures on the website, 93.9% for the diagnostic algorithm, 87.5% for the recommended further reading, 85.7% for the information document for patients and relatives and 70.0% for the checklist of the cleaning and disinfection of surfaces in contact with patients with CDI.

## Discussion

The training of health professionals is essential to the success of programs for the prevention of nosocomial infections, including CDI [8]. Previous research has reported a lack of knowledge of CDI among healthcare professionals [10].

In the present study, training provided as part of a program of CDI surveillance and control was associated with significant increases in the use of the most sensitive diagnostic algorithm and in the rate of CDI cases reported at hospitals participating in the VINCat program in Catalonia, and with greater compliance with diagnostic and preventive protocols. The training activity was also associated with a rise in the number of hospitals participating in the CDI surveillance program, probably due to the participation of the hospital infection control teams in the training program.

The mean incidence of CDI reported in Catalonia in 2016 (3.41 cases/10 000 patient-days) is similar to the rates in other Mediterranean countries and lower than those reported in northern Europe [11–13]. In the present study, there was a significant increase in all three types of CDI from 2011 to 2016 – hospital-acquired, non-nosocomial healthcare-related and (above all) in community-acquired infections. This increase in the incidence of CDI has been reported in other studies and has been associated with the implementation of more sensitive diagnostic techniques [13]. In this regard, our analysis of the evolution of the incidence of CDI reported by hospitals which already used an optimal diagnostic algorithm in 2011 and presented an adequate search intensity showed no increase in incidence over the 6 years of the study, indicating that the increased incidence observed in the study as a whole is due to the improvements in the diagnostic methods recorded in most hospitals. The use of suboptimal diagnostic methods is a limiting factor in the diagnosis of CDI; in agreement with other studies [14], we found the mean incidence of CDI reported by hospitals that applied an optimal diagnostic methodology (with a two-or-three step algorithm) to be significantly higher than at hospitals that did not. The incorporation of PCR to the diagnostic algorithm increases the detection of cases, due to its greater sensitivity [12, 15], although it is important to restrict the study to liquid or semi-liquid stools (as our participating centres did) in order to avoid the diagnosis of CDI in asymptomatic patients carrying *C. difficile*.

The lack of clinical suspicion is another fundamental factor in CDI underdiagnoses, which may occur in as many as 25% of cases. In a multinational European study, when CDI was systematically investigated in all faecal samples from patients with diarrhoea referred to the Microbiology laboratory regardless of whether the physician had asked for the test, it was found that in 23% of the cases of detected CDI, the study of *C. difficile* had not been requested [7]. Probably, in these cases, clinicians had suspected other causes of diarrhoea. In our study, hospitals with adequate search intensity for nosocomial CDI reported a significantly higher incidence than the rest.

The incidence of CDI in patients from community health centres is little known and probably underdiagnosed, although these patients may be a source of transmission of infection in acute care hospitals [16]. The training carried out increased clinical suspicion (though the difference was not statistically significant) in patients referred from nursing homes or from community health centres.

The incidence of community-acquired CDI practically doubled during the period of the study, probably in relation to the increase in search intensity in patients with previous antibiotic treatment. However, a recent study found that only 36% of community-acquired CDIs had received antibiotics [17], and so other risk factors must be considered [18].

Early notification by the microbiologist, which was the norm at the vast majority of participating centres, has been associated with early initiation of antibiotic treatment and preventive measures [19].

Although only two-thirds of the hospitals had their own diagnostic and/or preventive protocols, all had preventive measures for the transmission of CDI. The high basal degree of adherence to the preventive measures made it difficult to obtain a statistically significant improvement in the course of the study. Nonetheless, there was a non-significant increase in the implementation of certain preventive measures, such as hand hygiene using soap and water instead of alcohol (since the latter has a lower activity in the presence of spores), and in the number of centres that considered discontinuation of proton pump inhibitor (PPI) treatment after CDI diagnosis, bearing in mind that the epidemiological association between PPI and CDI suggests that these agents should be withdrawn unless they are essential [20, 21].

Regarding the degree of satisfaction with the various components of the training program, the most valued was the online training course, in which a large number of health professionals participated. This was followed by the recommendations on precautions for preventing CDI in health centres and the feedback with information on global and per hospital CDI rates.

One possible limitation of the study is the use of a self-administered, non-anonymous survey instead of an external audit. Although a certain bias in some of the responses cannot be ruled out, the respondents were informed that the results of their centre would not be published individually, thus minimizing the risk of such a bias. Another limitation of the study is the small number of hospitals that participated in the surveys on adherence to diagnostic and control measures, which made it difficult to obtain statistically significant differences between the two surveys.

In conclusion, the application of a multimodal training strategy was associated with an increase in the application of the optimal diagnostic algorithm, a significant rise in the reporting of CDI, and an increase in the number of hospitals participating in the VINCat program in Catalonia.
